# Rotheln Rubeola—German Measles

**Published:** 1878-06

**Authors:** 


ROTHELN, RUBEOLA—GERMAN MEASLES.—The two last preceding meetings of the Society having been favored by very able reports upon dermatology, by Prof. Yandell, considered in general and somewhat in detail, it will be the purpose of your committee at this session to call the attention of the profession to an epedemic exanthem that has been more or less prevalent throughout the State during the last year, and whose behavior and characteristics I have been enabled recently to observe. I allude to rubeola rotheln, or German measles, as it is more popularly called.

Unfortunately for our nomenclature, never very exact or praiseworthy, much confusion is likely to spring from adopting the latest appellation given to this exotic efflorescence, rubeola, by which title it is described by Dr. Thomas in Ziemssen's admirable work. As all English and American physicians have become familiar with that as the proper cognomen of measles, and any writing in our language which produces the term rubeola will most certainly carry the reader's mind to a consideration of measles; and as it is with that malady that the disease under consideration is most likely to be clinically confounded, it is doubly to be regretted that the nomenclature should also be identical; for unlimited confusion must necessarily ensue, when trying to unravel and classify a doubtful case, to find different phenomena described as belonging to identical names, or the reverse. The only other classical designation, rotheln, is almost unpronounceable by other than a German tongue; and the vulgar cognomen, while sufficient for family use, will hardly be acceptable to our more fastidious faculty. While disclaiming any hope of meeting a second in my suggestion, I think I will venture to propose that a happy solution of this difficulty could have been found, before the publication of Ziemssen's Cyclopedia, in the adoption of the term "*rubella*" (as has already been applied to this or a similar eruption) as a name carrying with it the significance of *diminutive;* then rubella would signify *littte measles*—just the idea that best describes in a brief word the disease in question.

Not until the middle of the last century was any attempt made to eliminate the phenomena of rotheln from those of measles, scarlatina, and roseola. German writers were the first to begin to bring some order out of the chaos surrounding these perplexing exanthems, and the clearest and most satisfactory works upon the subject are by German authors. English writers are yet exceedingly muddled and perplexed, and give no clearly defined description of this disease. Mr. Tilbury Fox, who has written an exceedingly good and useful book for the busy practitioner, is entirely at sea in regard to the distinctive entity of rotheln, and describes the general characteristics of the disease in half a dozen different places in his book, and under a multiplicity of names, and confounds probably several distinct diseases. In our day, and in our own country, a great variety of opinion exists, and a good many pages have been written, attempting to give the disease its proper status as a distinct variety of acute exanthem, instead of the erroneous idea that it was a hybrid or bastard form of measles or scarlatina.

These two last-named diseases are the ones with which rotheln has been most often confounded; the reasons for difference of opinion as to which of these diseases it most resembles being accidental, and will be pointed out further along.

Ignorant or designing persons have taken advantage of this resemblance to these forms of grave disease to claim great proficiency in the treatment of scarlet fever, having in hand great numbers of cases of this mild and insignificant exanthem, and claiming their recoveries as so many cases saved from death from scarlatina.

An epidemic of rotheln is at this time prevalent in my immediate vicinity, and I will give here the following clincal history.

After an uncertain period of incubation, covering probably a fortnight, without any very noticeable prodromata, an eruption becomes visible on the face and neck, and quickly spreads over the chest and to the extremities. This reaches its climax in about twenty-four to forty-eight hours, and occupies about the same period in taking its leave, the parts first "breaking up" being the first to recover. In many cases the eruption "comes and goes," to use a very homely and a very expressive term; at times being almost entirely invisible on any part of the body, and in a little while the whole surface will be thickly covered. The most noticeable feature within my observation in regard to the eruption itself, is the fact that in nearly if not quite all the cases, the eruption has a *fady* appearance, *as if it were subcuticular;* looking as though a thin pellicle covered it, much like measles in its early stages, before the eruption is well out. This is characteristic throughout the course of the disease. As it is in the beginning, so at the climax; in other words, not "coming out," like measles, to a bright blush. This renders the rash much less bright in color, and less prominent as a feature of the disease than the rash of measles. Again, unlike measles, I have met with no case that the eruption could be felt above the cuticular surface, as in measles, the patches being under the surface, and simply a discoloration and not an elevation.

In no case have I met with any *desquamation*, though it is possible in an exceedingly severe form the cuticular inflammation may run so high, or a vesicular patch be produced which might give rise to a slight death of cuticle.

*Glandular Swellings.*—About the second day, or the period of climax of eruption, the *submaxillary* and *cervical glands* become inflamed and swollen, often to a very great degree, forming the most painful feature of the disease. The parotids will at times also become more or less affected, but the most frequently-sized and greatest sufferers are submaxillary. These remain swollen and tender, generally several days after the disappearance of any other symptom.

*Buccal Symptoms.*—As early as the first or second day the buccal mucous lining becomes inflamed, and ulcerations appear on the gums, tip of tongue, and the lips. Frequently the lips become enormously swollen, and the whole mucous border covered by ulcerations; and in a few days thick, black, or yellowish crusts render the taking of food or drink painful and difficult. The tonsils now and then are a little sore and swollen, but this rarely occurs. I have not seen a patient who had "sore throat," but get histories in a few instances of pharyngeal inflammation of a light character, generally prodromatous, however, and not as an accompaniment or sequel, as in scarlet fever.

*Tongue.*—From the beginning of the eruption until the period of decline, in the worst cases, the tongue is covered by a very dense, long, yellowish brown epithelium, but is not dry, nor does the color get darker. The end is blistered and the edges red.

These are the more prominent general symptoms, and is a picture of the most severe forms of the disease. A very large percentage escape with these symptoms scarcely more than shadowed, and the patients rarely go to bed, and seldom think it necessary to send for a physician. *In only the worst forms of the disease is the temperature appreciably elevated.* I have never found it over 100.2°, and that only in one case.

*Anomalous Variety.*—In a few instances I have met with cases giving all the symptoms detailed above, *but without any eruption.* In these cases the cheeks and forehead are of the deepest and darkest red hue; not the bright scarlet of scarlet fever, but a rich *mahogany crimson* (if I may invent a shade). Some whole families have this form of disease, and occasionally both forms will appear in the same family. Within my observation all the cases of this anomalous form of the disease were in patients with dark hair and eyes—brunettes, in other words. It is from this form, doubtless, that rotheln has been confounded with scarlatina, as these cases all had the glandular and buccal symptoms in their worst forms.

*Differential Diagnosis.*—1. From either scarlatina or measles: the absence generally of a prodromata, and when they occur in their light form.

2. From measles: the eruption in rotheln is not raised above the cuticular surface, looks fady, and as if it were under the cuticle.

3. From scarlatina: by the absence of the punctate form of eruption, the crimson blush being general over the face, and confined to that surface—as I have not been able to see the deep blush under the clothing; also by the absence of the anginose symptoms so marked in scarlatina; and finally by the want of desquamation, renal, or other sequelæ.

4. The severe buccal symptoms, the ulcerated tongue and lips, the thick, angry-looking crusts, and submaxillary glands are more pronounced in bad cases than in either measles or scarlatina.

*The critical Test of the Thermometer.*—The temperature is seldom above the normal—never more than 100.5°; whereas in measles it will reach 104° or 105°, and in scarlatina it may even attain 106°.

6. The shorter course of the disease: the period of eruption only lasting from two to four days, and the rapid and entire recovery without sequelæ.

7. These diseases are not severally naturally protective, an attack of one affording no immunity from either of the others. At this time I have charge of a family of seven persons who have gone through an epidemic of rotheln, each of whom had previously had measles.

*Treatment.*—Rotheln requires no special treatment. In the vast majority of cases a physician is not called. The disease runs a light and brief course, and only requires simple medication for accidental complications—a mild purgative and some simple mouth-wash.—*Louisville Medical News.*

